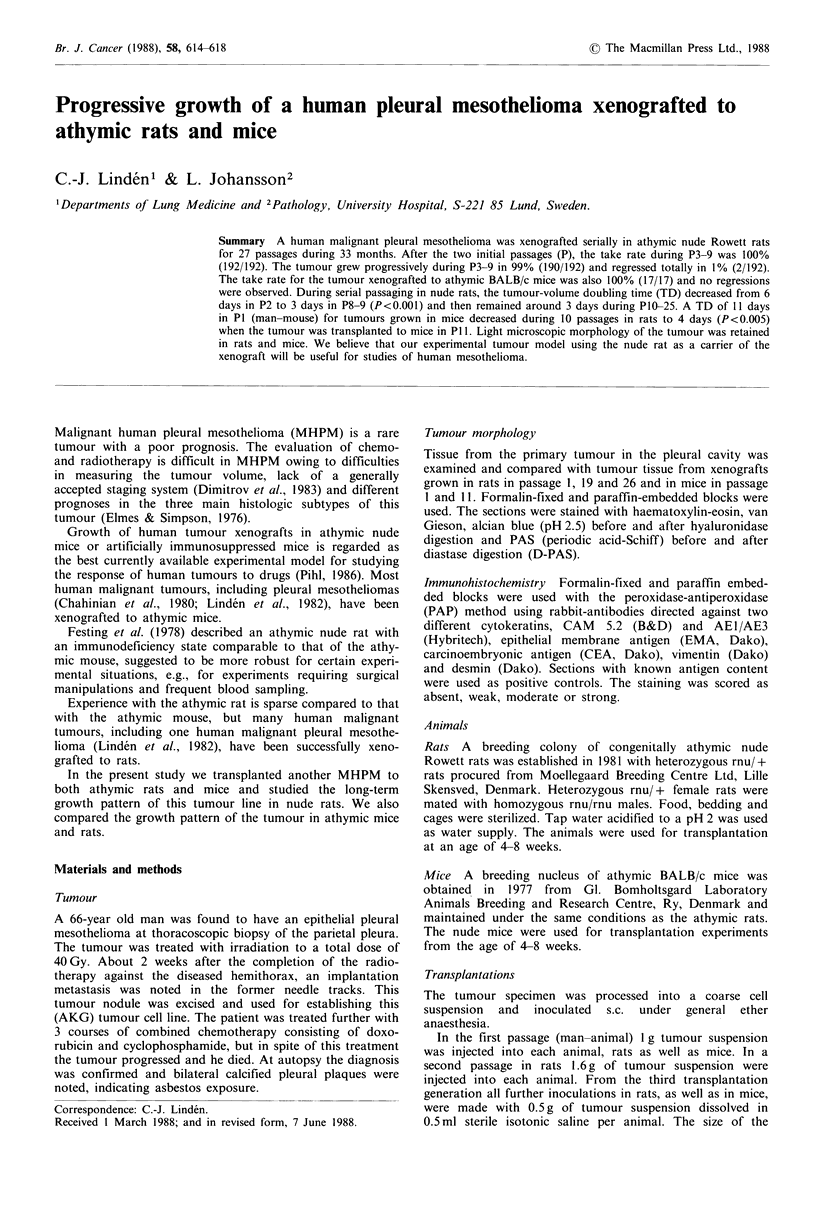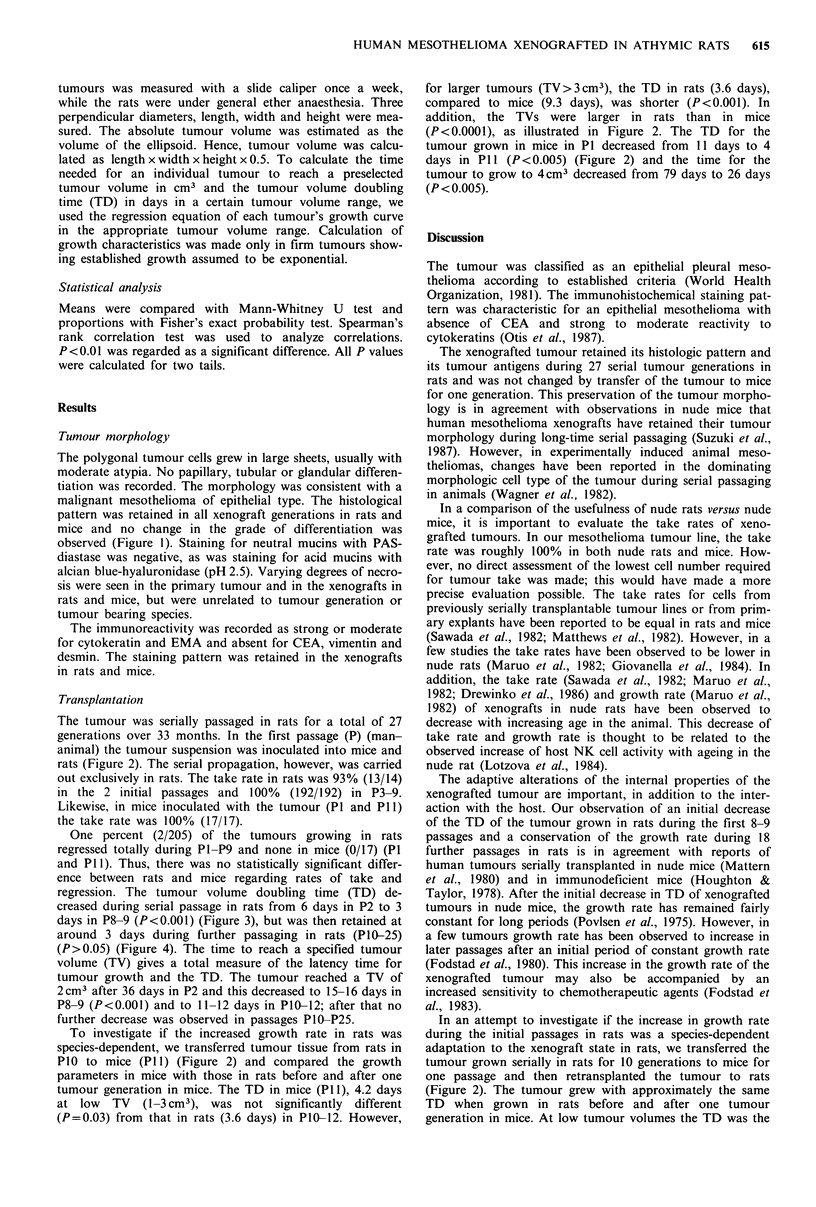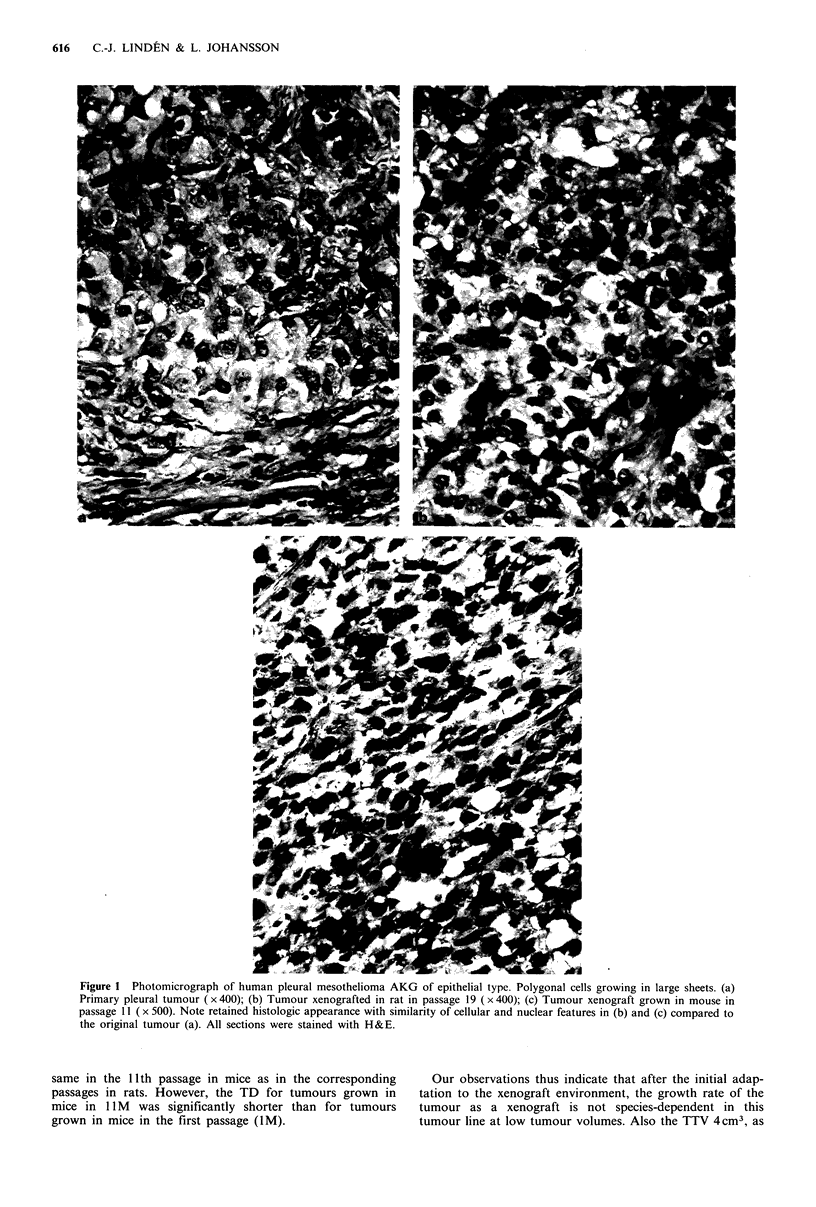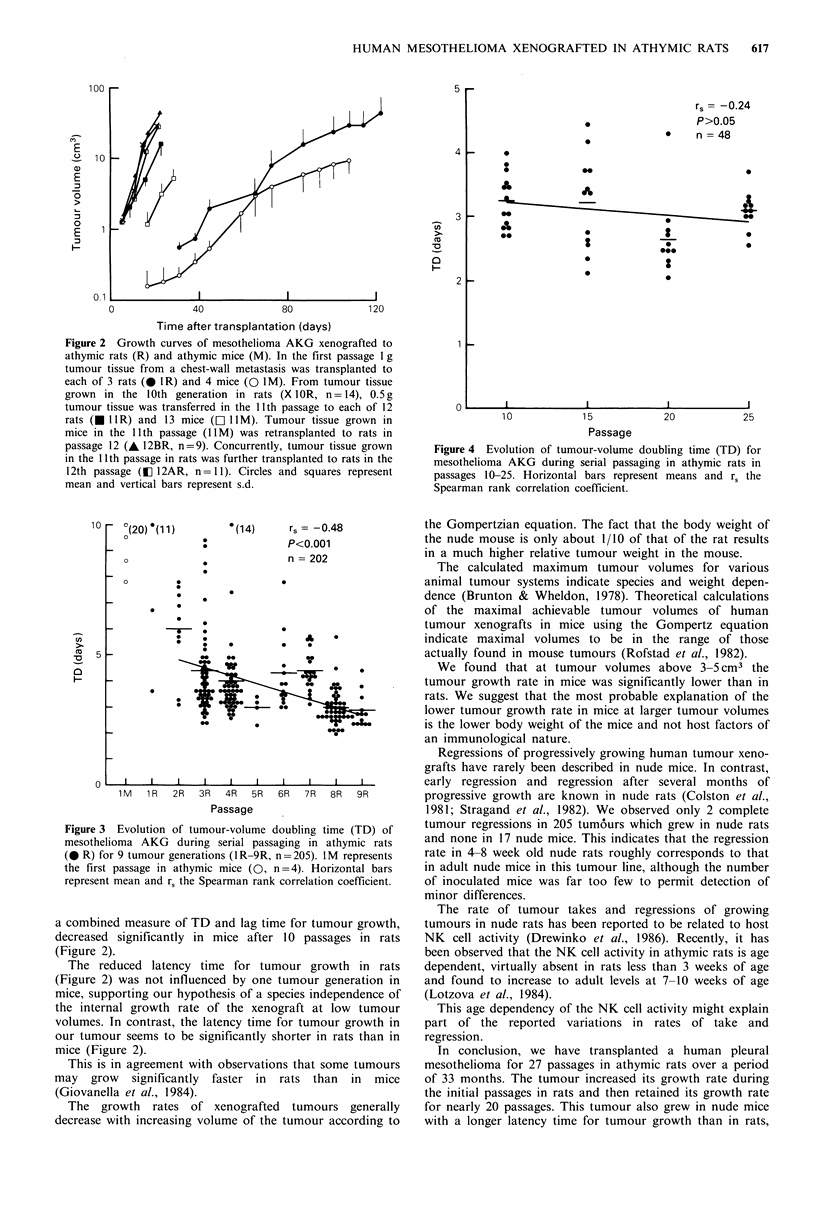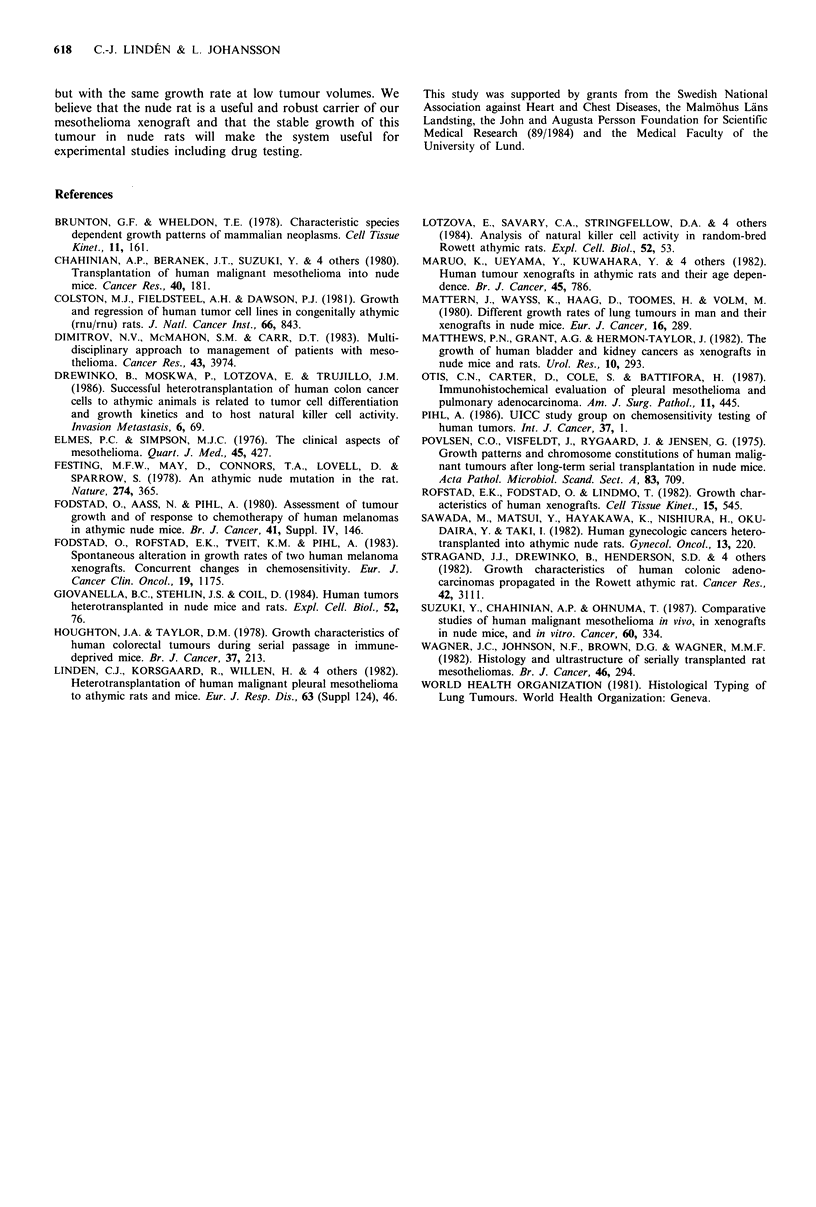# Progressive growth of a human pleural mesothelioma xenografted to athymic rats and mice.

**DOI:** 10.1038/bjc.1988.270

**Published:** 1988-11

**Authors:** C. J. LindÃ©n, L. Johansson

**Affiliations:** DÃ¨partment of Lung Medicine, University Hospital, Lund, Sweden.

## Abstract

**Images:**


© The Macmillan Press Ltd., 1988

### Progressive growth of a human pleural mesothelioma xenografted to athymic rats and mice

C.-J. Lindén[1] & L. Johansson[2]

[1]Departments of Lung Medicine and [2]Pathology, University Hospital, S-221 85 Lund, Sweden.

**Summary** A human malignant pleural mesothelioma was xenografted serially in athymic nude Rowett rats for 27 passages during 33 months. After the two initial passages (P), the take rate during P3–9 was 100% (192/192). The tumour grew progressively during P3–9 in 99% (190/192) and regressed totally in 1% (2/192). The take rate for the tumour xenografted to athymic BALB/c mice was also 100% (17/17) and no regressions were observed. During serial passaging in nude rats, the tumour-volume doubling time (TD) decreased from 6 days in P2 to 3 days in P8–9 ($P<0.001$) and then remained around 3 days during P10–25. A TD of 11 days in P1 (man-mouse) for tumours grown in mice decreased during 10 passages in rats to 4 days ($P<0.005$) when the tumour was transplanted to mice in P11. Light microscopic morphology of the tumour was retained in rats and mice. We believe that our experimental tumour model using the nude rat as a carrier of the xenograft will be useful for studies of human mesothelioma.

Malignant human pleural mesothelioma (MHPM) is a rare tumour with a poor prognosis. The evaluation of chemo- and radiotherapy is difficult in MHPM owing to difficulties in measuring the tumour volume, lack of a generally accepted staging system (Dimitrov *et al.*, 1983) and different prognoses in the three main histologic subtypes of this tumour (Elmes & Simpson, 1976).

Growth of human tumour xenografts in athymic nude mice or artificially immunosuppressed mice is regarded as the best currently available experimental model for studying the response of human tumours to drugs (Pihl, 1986). Most human malignant tumours, including pleural mesotheliomas (Chahinian *et al.*, 1980; Lindén *et al.*, 1982), have been xenografted to athymic mice.

Festing *et al.* (1978) described an athymic nude rat with an immunodeficiency state comparable to that of the athymic mouse, suggested to be more robust for certain experimental situations, e.g., for experiments requiring surgical manipulations and frequent blood sampling.

Experience with the athymic rat is sparse compared to that with the athymic mouse, but many human malignant tumours, including one human malignant pleural mesothelioma (Lindén *et al.*, 1982), have been successfully xenografted to rats.

In the present study we transplanted another MHPM to both athymic rats and mice and studied the long-term growth pattern of this tumour line in nude rats. We also compared the growth pattern of the tumour in athymic mice and rats.

### Materials and methods

### *Tumour*

A 66-year old man was found to have an epithelial pleural mesothelioma at thoracoscopic biopsy of the parietal pleura. The tumour was treated with irradiation to a total dose of 40 Gy. About 2 weeks after the completion of the radiotherapy against the diseased hemithorax, an implantation metastasis was noted in the former needle tracks. This tumour nodule was excised and used for establishing this (AKG) tumour cell line. The patient was treated further with 3 courses of combined chemotherapy consisting of doxorubicin and cyclophosphamide, but in spite of this treatment the tumour progressed and he died. At autopsy the diagnosis was confirmed and bilateral calcified pleural plaques were noted, indicating asbestos exposure.

Correspondence: C.-J. Lindén.

Received 1 March 1988; and in revised form, 7 June 1988.

### *Tumour morphology*

Tissue from the primary tumour in the pleural cavity was examined and compared with tumour tissue from xenografts grown in rats in passage 1, 19 and 26 and in mice in passage 1 and 11. Formalin-fixed and paraffin-embedded blocks were used. The sections were stained with haematoxylin-eosin, van Gieson, alcian blue (pH 2.5) before and after hyaluronidase digestion and PAS (periodic acid-Schiff) before and after diastase digestion (D-PAS).

*Immunohistochemistry* Formalin-fixed and paraffin embedded blocks were used with the peroxidase-antiperoxidase (PAP) method using rabbit-antibodies directed against two different cytokeratins, CAM 5.2 (B&D) and AE1/AE3 (Hybritech), epithelial membrane antigen (EMA, Dako), carcinoembryonic antigen (CEA, Dako), vimentin (Dako) and desmin (Dako). Sections with known antigen content were used as positive controls. The staining was scored as absent, weak, moderate or strong.

### *Animals*

*Rats* A breeding colony of congenitally athymic nude Rowett rats was established in 1981 with heterozygous rnu/+ rats procured from Moellegaard Breeding Centre Ltd, Lille Skensved, Denmark. Heterozygous rnu/+ female rats were mated with homozygous rnu/rnu males. Food, bedding and cages were sterilized. Tap water acidified to a pH 2 was used as water supply. The animals were used for transplantation at an age of 4–8 weeks.

*Mice* A breeding nucleus of athymic BALB/c mice was obtained in 1977 from Gl. Bomholtsgard Laboratory Animals Breeding and Research Centre, Ry, Denmark and maintained under the same conditions as the athymic rats. The nude mice were used for transplantation experiments from the age of 4–8 weeks.

### *Transplantations*

The tumour specimen was processed into a coarse cell suspension and inoculated s.c. under general ether anaesthesia.

In the first passage (man–animal) 1 g tumour suspension was injected into each animal, rats as well as mice. In a second passage in rats 1.6 g of tumour suspension were injected into each animal. From the third transplantation generation all further inoculations in rats, as well as in mice, were made with 0.5 g of tumour suspension dissolved in 0.5 ml sterile isotonic saline per animal. The size of the tumours was measured with a slide caliper once a week, while the rats were under general ether anaesthesia. Three perpendicular diameters, length, width and height were measured. The absolute tumour volume was estimated as the volume of the ellipsoid. Hence, tumour volume was calculated as length × width × height × 0.5. To calculate the time needed for an individual tumour to reach a preselected tumour volume in $cm^3$ and the tumour volume doubling time (TD) in days in a certain tumour volume range, we used the regression equation of each tumour's growth curve in the appropriate tumour volume range. Calculation of growth characteristics was made only in firm tumours showing established growth assumed to be exponential.

### *Statistical analysis*

Means were compared with Mann-Whitney U test and proportions with Fisher's exact probability test. Spearman's rank correlation test was used to analyze correlations. $P<0.01$ was regarded as a significant difference. All $P$ values were calculated for two tails.

### Results

### *Tumour morphology*

The polygonal tumour cells grew in large sheets, usually with moderate atypia. No papillary, tubular or glandular differentiation was recorded. The morphology was consistent with a malignant mesothelioma of epithelial type. The histological pattern was retained in all xenograft generations in rats and mice and no change in the grade of differentiation was observed (Figure 1). Staining for neutral mucins with PAS-diastase was negative, as was staining for acid mucins with alcian blue-hyaluronidase (pH 2.5). Varying degrees of necrosis were seen in the primary tumour and in the xenografts in rats and mice, but were unrelated to tumour generation or tumour bearing species.

The immunoreactivity was recorded as strong or moderate for cytokeratin and EMA and absent for CEA, vimentin and desmin. The staining pattern was retained in the xenografts in rats and mice.

### *Transplantation*

The tumour was serially passaged in rats for a total of 27 generations over 33 months. In the first passage (P) (man-animal) the tumour suspension was inoculated into mice and rats (Figure 2). The serial propagation, however, was carried out exclusively in rats. The take rate in rats was 93% (13/14) in the 2 initial passages and 100% (192/192) in P3–9. Likewise, in mice inoculated with the tumour (P1 and P11) the take rate was 100% (17/17).

One percent (2/205) of the tumours growing in rats regressed totally during P1–P9 and none in mice (0/17) (P1 and P11). Thus, there was no statistically significant difference between rats and mice regarding rates of take and regression. The tumour volume doubling time (TD) decreased during serial passage in rats from 6 days in P2 to 3 days in P8–9 ($P<0.001$) (Figure 3), but was then retained at around 3 days during further passaging in rats (P10–25) ($P>0.05$) (Figure 4). The time to reach a specified tumour volume (TV) gives a total measure of the latency time for tumour growth and the TD. The tumour reached a TV of $2\,cm^3$ after 36 days in P2 and this decreased to 15–16 days in P8–9 ($P<0.001$) and to 11–12 days in P10–12; after that no further decrease was observed in passages P10–P25.

To investigate if the increased growth rate in rats was species-dependent, we transferred tumour tissue from rats in P10 to mice (P11) (Figure 2) and compared the growth parameters in mice with those in rats before and after one tumour generation in mice. The TD in mice (P11), 4.2 days at low TV (1–$3\,cm^3$), was not significantly different ($P=0.03$) from that in rats (3.6 days) in P10–12. However, for larger tumours (TV > $3\,cm^3$), the TD in rats (3.6 days), compared to mice (9.3 days), was shorter ($P<0.001$). In addition, the TVs were larger in rats than in mice ($P<0.0001$), as illustrated in Figure 2. The TD for the tumour grown in mice in P1 decreased from 11 days to 4 days in P11 ($P<0.005$) (Figure 2) and the time for the tumour to grow to $4\,cm^3$ decreased from 79 days to 26 days ($P<0.005$).

### Discussion

The tumour was classified as an epithelial pleural mesothelioma according to established criteria (World Health Organization, 1981). The immunohistochemical staining pattern was characteristic for an epithelial mesothelioma with absence of CEA and strong to moderate reactivity to cytokeratins (Otis *et al.*, 1987).

The xenografted tumour retained its histologic pattern and its tumour antigens during 27 serial tumour generations in rats and was not changed by transfer of the tumour to mice for one generation. This preservation of the tumour morphology is in agreement with observations in nude mice that human mesothelioma xenografts have retained their tumour morphology during long-time serial passaging (Suzuki *et al.*, 1987). However, in experimentally induced animal mesotheliomas, changes have been reported in the dominating morphologic cell type of the tumour during serial passaging in animals (Wagner *et al.*, 1982).

In a comparison of the usefulness of nude rats *versus* nude mice, it is important to evaluate the take rates of xenografted tumours. In our mesothelioma tumour line, the take rate was roughly 100% in both nude rats and mice. However, no direct assessment of the lowest cell number required for tumour take was made; this would have made a more precise evaluation possible. The take rates for cells from previously serially transplantable tumour lines or from primary explants have been reported to be equal in rats and mice (Sawada *et al.*, 1982; Matthews *et al.*, 1982). However, in a few studies the take rates have been observed to be lower in nude rats (Maruo *et al.*, 1982; Giovanella *et al.*, 1984). In addition, the take rate (Sawada *et al.*, 1982; Maruo *et al.*, 1982; Drewinko *et al.*, 1986) and growth rate (Maruo *et al.*, 1982) of xenografts in nude rats have been observed to decrease with increasing age in the animal. This decrease of take rate and growth rate is thought to be related to the observed increase of host NK cell activity with ageing in the nude rat (Lotzova *et al.*, 1984).

The adaptive alterations of the internal properties of the xenografted tumour are important, in addition to the interaction with the host. Our observation of an initial decrease of the TD of the tumour grown in rats during the first 8–9 passages and a conservation of the growth rate during 18 further passages in rats is in agreement with reports of human tumours serially transplanted in nude mice (Mattern *et al.*, 1980) and in immunodeficient mice (Houghton & Taylor, 1978). After the initial decrease in TD of xenografted tumours in nude mice, the growth rate has remained fairly constant for long periods (Povlsen *et al.*, 1975). However, in a few tumours growth rate has been observed to increase in later passages after an initial period of constant growth rate (Fodstad *et al.*, 1980). This increase in the growth rate of the xenografted tumour may also be accompanied by an increased sensitivity to chemotherapeutic agents (Fodstad *et al.*, 1983).

In an attempt to investigate if the increase in growth rate during the initial passages in rats was a species-dependent adaptation to the xenograft state in rats, we transferred the tumour grown serially in rats for 10 generations to mice for one passage and then retransplanted the tumour to rats (Figure 2). The tumour grew with approximately the same TD when grown in rats before and after one tumour generation in mice. At low tumour volumes the TD was the

**Figure 1** Photomicrograph of human pleural mesothelioma AKG of epithelial type. Polygonal cells growing in large sheets. (a) Primary pleural tumour (×400); (b) Tumour xenografted in rat in passage 19 (×400); (c) Tumour xenograft grown in mouse in passage 11 (×500). Note retained histologic appearance with similarity of cellular and nuclear features in (b) and (c) compared to the original tumour (a). All sections were stained with H&E.

same in the 11th passage in mice as in the corresponding passages in rats. However, the TD for tumours grown in mice in 11M was significantly shorter than for tumours grown in mice in the first passage (1M).

Our observations thus indicate that after the initial adaptation to the xenograft environment, the growth rate of the tumour as a xenograft is not species-dependent in this tumour line at low tumour volumes. Also the TTV 4 $cm^3$, as

**Figure 2** Growth curves of mesothelioma AKG xenografted to athymic rats (R) and athymic mice (M). In the first passage 1 g tumour tissue from a chest-wall metastasis was transplanted to each of 3 rats (● 1R) and 4 mice (○ 1M). From tumour tissue grown in the 10th generation in rats (X10R, n = 14), 0.5 g tumour tissue was transferred in the 11th passage to each of 12 rats (■ 11R) and 13 mice (□ 11M). Tumour tissue grown in mice in the 11th passage (11M) was retransplanted to rats in passage 12 (▲ 12BR, n = 9). Concurrently, tumour tissue grown in the 11th passage in rats was further transplanted to rats in the 12th passage (◧ 12AR, n = 11). Circles and squares represent mean and vertical bars represent s.d.

**Figure 4** Evolution of tumour-volume doubling time (TD) for mesothelioma AKG during serial passaging in athymic rats in passages 10–25. Horizontal bars represent means and $r_s$ the Spearman rank correlation coefficient.

**Figure 3** Evolution of tumour-volume doubling time (TD) of mesothelioma AKG during serial passaging in athymic rats (● R) for 9 tumour generations (1R–9R, n = 205). 1M represents the first passage in athymic mice (○, n = 4). Horizontal bars represent mean and $r_s$ the Spearman rank correlation coefficient.

a combined measure of TD and lag time for tumour growth, decreased significantly in mice after 10 passages in rats (Figure 2).

The reduced latency time for tumour growth in rats (Figure 2) was not influenced by one tumour generation in mice, supporting our hypothesis of a species independence of the internal growth rate of the xenograft at low tumour volumes. In contrast, the latency time for tumour growth in our tumour seems to be significantly shorter in rats than in mice (Figure 2).

This is in agreement with observations that some tumours may grow significantly faster in rats than in mice (Giovanella *et al.*, 1984).

The growth rates of xenografted tumours generally decrease with increasing volume of the tumour according to the Gompertzian equation. The fact that the body weight of the nude mouse is only about 1/10 of that of the rat results in a much higher relative tumour weight in the mouse.

The calculated maximum tumour volumes for various animal tumour systems indicate species and weight dependence (Brunton & Wheldon, 1978). Theoretical calculations of the maximal achievable tumour volumes of human tumour xenografts in mice using the Gompertz equation indicate maximal volumes to be in the range of those actually found in mouse tumours (Rofstad *et al.*, 1982).

We found that at tumour volumes above 3–5 cm$^3$ the tumour growth rate in mice was significantly lower than in rats. We suggest that the most probable explanation of the lower tumour growth rate in mice at larger tumour volumes is the lower body weight of the mice and not host factors of an immunological nature.

Regressions of progressively growing human tumour xenografts have rarely been described in nude mice. In contrast, early regression and regression after several months of progressive growth are known in nude rats (Colston *et al.*, 1981; Stragand *et al.*, 1982). We observed only 2 complete tumour regressions in 205 tumours which grew in nude rats and none in 17 nude mice. This indicates that the regression rate in 4–8 week old nude rats roughly corresponds to that in adult nude mice in this tumour line, although the number of inoculated mice was far too few to permit detection of minor differences.

The rate of tumour takes and regressions of growing tumours in nude rats has been reported to be related to host NK cell activity (Drewinko *et al.*, 1986). Recently, it has been observed that the NK cell activity in athymic rats is age dependent, virtually absent in rats less than 3 weeks of age and found to increase to adult levels at 7–10 weeks of age (Lotzova *et al.*, 1984).

This age dependency of the NK cell activity might explain part of the reported variations in rates of take and regression.

In conclusion, we have transplanted a human pleural mesothelioma for 27 passages in athymic rats over a period of 33 months. The tumour increased its growth rate during the initial passages in rats and then retained its growth rate for nearly 20 passages. This tumour also grew in nude mice with a longer latency time for tumour growth than in rats, but with the same growth rate at low tumour volumes. We believe that the nude rat is a useful and robust carrier of our mesothelioma xenograft and that the stable growth of this tumour in nude rats will make the system useful for experimental studies including drug testing.

This study was supported by grants from the Swedish National Association against Heart and Chest Diseases, the Malmöhus Läns Landsting, the John and Augusta Persson Foundation for Scientific Medical Research (89/1984) and the Medical Faculty of the University of Lund.

### References

BRUNTON, G.F. & WHELDON, T.E. (1978). Characteristic species dependent growth patterns of mammalian neoplasms. *Cell Tissue Kinet.*, **11**, 161.

CHAHINIAN, A.P., BERANEK, J.T., SUZUKI, Y. & 4 others (1980). Transplantation of human malignant mesothelioma into nude mice. *Cancer Res.*, **40**, 181.

COLSTON, M.J., FIELDSTEEL, A.H. & DAWSON, P.J. (1981). Growth and regression of human tumor cell lines in congenitally athymic (rnu/rnu) rats. *J. Natl. Cancer Inst.*, **66**, 843.

DIMITROV, N.V., McMAHON, S.M. & CARR, D.T. (1983). Multidisciplinary approach to management of patients with mesothelioma. *Cancer Res.*, **43**, 3974.

DREWINKO, B., MOSKWA, P., LOTZOVA, E. & TRUJILLO, J.M. (1986). Successful heterotransplantation of human colon cancer cells to athymic animals is related to tumor cell differentiation and growth kinetics and to host natural killer cell activity. *Invasion Metastasis*, **6**, 69.

ELMES, P.C. & SIMPSON, M.J.C. (1976). The clinical aspects of mesothelioma. *Quart. J. Med.*, **45**, 427.

FESTING, M.F.W., MAY, D., CONNORS, T.A., LOVELL, D. & SPARROW, S. (1978). An athymic nude mutation in the rat. *Nature*, **274**, 365.

FODSTAD, O., AASS, N. & PIHL, A. (1980). Assessment of tumour growth and of response to chemotherapy of human melanomas in athymic nude mice. *Br. J. Cancer*, **41**, Suppl. IV, 146.

FODSTAD, O., ROFSTAD, E.K., TVEIT, K.M. & PIHL, A. (1983). Spontaneous alteration in growth rates of two human melanoma xenografts. Concurrent changes in chemosensitivity. *Eur. J. Cancer Clin. Oncol.*, **19**, 1175.

GIOVANELLA, B.C., STEHLIN, J.S. & COIL, D. (1984). Human tumors heterotransplanted in nude mice and rats. *Expl. Cell. Biol.*, **52**, 76.

HOUGHTON, J.A. & TAYLOR, D.M. (1978). Growth characteristics of human colorectal tumours during serial passage in immune-deprived mice. *Br. J. Cancer*, **37**, 213.

LINDEN, C.J., KORSGAARD, R., WILLEN, H. & 4 others (1982). Heterotransplantation of human malignant pleural mesothelioma to athymic rats and mice. *Eur. J. Resp. Dis.*, **63** (Suppl 124), 46.

LOTZOVA, E., SAVARY, C.A., STRINGFELLOW, D.A. & 4 others (1984). Analysis of natural killer cell activity in random-bred Rowett athymic rats. *Expl. Cell. Biol.*, **52**, 53.

MARUO, K., UEYAMA, Y., KUWAHARA, Y. & 4 others (1982). Human tumour xenografts in athymic rats and their age dependence. *Br. J. Cancer*, **45**, 786.

MATTERN, J., WAYSS, K., HAAG, D., TOOMES, H. & VOLM, M. (1980). Different growth rates of lung tumours in man and their xenografts in nude mice. *Eur. J. Cancer*, **16**, 289.

MATTHEWS, P.N., GRANT, A.G. & HERMON-TAYLOR, J. (1982). The growth of human bladder and kidney cancers as xenografts in nude mice and rats. *Urol. Res.*, **10**, 293.

OTIS, C.N., CARTER, D., COLE, S. & BATTIFORA, H. (1987). Immunohistochemical evaluation of pleural mesothelioma and pulmonary adenocarcinoma. *Am. J. Surg. Pathol.*, **11**, 445.

PIHL, A. (1986). UICC study group on chemosensitivity testing of human tumors. *Int. J. Cancer*, **37**, 1.

POVLSEN, C.O., VISFELDT, J., RYGAARD, J. & JENSEN, G. (1975). Growth patterns and chromosome constitutions of human malignant tumours after long-term serial transplantation in nude mice. *Acta Pathol. Microbiol. Scand. Sect. A*, **83**, 709.

ROFSTAD, E.K., FODSTAD, O. & LINDMO, T. (1982). Growth characteristics of human xenografts. *Cell Tissue Kinet.*, **15**, 545.

SAWADA, M., MATSUI, Y., HAYAKAWA, K., NISHIURA, H., OKUDAIRA, Y. & TAKI, I. (1982). Human gynecologic cancers heterotransplanted into athymic nude rats. *Gynecol. Oncol.*, **13**, 220.

STRAGAND, J.J., DREWINKO, B., HENDERSON, S.D. & 4 others (1982). Growth characteristics of human colonic adenocarcinomas propagated in the Rowett athymic rat. *Cancer Res.*, **42**, 3111.

SUZUKI, Y., CHAHINIAN, A.P. & OHNUMA, T. (1987). Comparative studies of human malignant mesothelioma *in vivo*, in xenografts in nude mice, and *in vitro*. *Cancer*, **60**, 334.

WAGNER, J.C., JOHNSON, N.F., BROWN, D.G. & WAGNER, M.M.F. (1982). Histology and ultrastructure of serially transplanted rat mesotheliomas. *Br. J. Cancer*, **46**, 294.

WORLD HEALTH ORGANIZATION (1981). Histological Typing of Lung Tumours. World Health Organization: Geneva.